# Application of the C3-Binding Motif of Streptococcal Pyrogenic Exotoxin B to Protect Mice from Invasive Group A Streptococcal Infection

**DOI:** 10.1371/journal.pone.0117268

**Published:** 2015-01-28

**Authors:** Chih-Feng Kuo, Nina Tsao, Miao-Hui Cheng, Hsiu-Chen Yang, Yu-Chieh Wang, Ying-Pin Chen, Kai-Jen Lin

**Affiliations:** 1 Department of Nursing, I-Shou University, Kaohsiung City, Taiwan; 2 Department of Biological Science and Technology, I-Shou University, Kaohsiung City, Taiwan; 3 Department of Pathology, E-DA Hospital, Kaohsiung City, Taiwan; Wake Forest University School of Medicine, UNITED STATES

## Abstract

Group A streptococcus (GAS) is an important human pathogen that produces several extracellular exotoxins to facilitate invasion and infection. Streptococcal pyrogenic exotoxin B (SPE B) has been demonstrated to be an important virulence factor of GAS. Our previous studies indicate that SPE B cleaves complement 3 (C3) and inhibits the activation of complement pathways. In this study, we constructed and expressed recombinant fragments of SPE B to examine the C3-binding site of SPE B. Using enzyme-linked immunosorbent assays and pull-down assays, we found that the C-terminal domain, containing amino-acid residues 345–398, of SPE B was the major binding site of human serum C3. We further identified a major, Ala^376^-Pro^398^, and a minor C3-binding motif, Gly^346^-Gly^360^, that both mediated the binding of C3 complement. Immunization with the C3-binding motifs protected mice against challenge with a lethal dose of non-invasive M49 strain GAS but not invasive M1 strains. To achieve higher efficiency against invasive M1 GAS infection, a combination of synthetic peptides derived from C-terminal epitope of streptolysin S (SLSpp) and from the major C3-binding motif of SPE B (PP6, Ala^376^-Pro^398^) was used to elicit specific immune response to those two important streptococcal exotoxins. Death rates and the severity of skin lesions decreased significantly in PP6/SLSpp-immunized mice that were infected with invasive M1 strains of GAS. These results indicate a combination of the C3-binding motif of SPE B and the protective epitope of SLS could be used as a subunit vaccine against invasive M1 strains group A streptococcal infection.

## Introduction

Group A streptococcus (GAS, *Streptococcus pyogenes*) causes pharyngitis, tonsillitis, cellulitis, scarlet fever, myositis, necrotizing fasciitis, puerperal sepsis, and streptococcal toxic shock syndrome. Post-streptococcal glomerulonephritis and rheumatic fever are serious post-infectious immune sequelae following scarlet fever or repeated GAS infection [1, 2, 3]. The GAS-mediated disease severity is associated with bacterial surface virulence factors, such as hyaluronic acid capsules and M proteins, and secreted exotoxins, including C5a peptidase, streptolysin S (SLS), streptolysin O (SLO) and streptococcal pyrogenic exotoxins (SPEs) [4, 5]. There are numbers of vaccine candidates against GAS infection in clinical and preclinical development, including multivalent N-terminal type-specific M protein, the conserved epitope in the C-terminal region of the M protein, fibronectin-binding protein, and C5a peptidase [6, 7]. However, the group A streptococcal clinical vaccines has not been proven to protect against GAS infection.

Previous studies suggest that the streptococcal pyrogenic exotoxin B (SPE B), an extracellular cysteine protease, contributes to increasing GAS invasion and infection [8, 9]. SPE B produced from GAS is a 42-kDa zymogen that is autoprocessed to a 28-kDa mature form. The mature form of SPE B can degrade several host proteins, including fibronectin, vitronectin, matrix metalloprotease, protease-activated receptor-1, occludin, and E-cadherin which play an important role in GAS pathogenesis [10, 11, 12, 13].

The complement system plays an essential role in the early phase of the host defense against bacterial infection. Three different complement pathways converge in the generation of complement 3 (C3) convertase to cleave C3 to activate the complement cascades. Activation of C3 plays a key role in eliminating bacteria and initiates the common pathway, although the initiation stages in the classical, lectin and alternative complement pathways are diverse. GAS uses several virulence factors to block the activation of complement and prevent complement-mediated killing, including M protein, protein H, C5a peptidase, streptococcus inhibitor of complement (SIC) and SPE B [14, 15, 16, 17, 18].

In our previous study, we found that SPE B could effectively bind and degrade properdin and C3 and led to a decrease of opsonophagocytosis-mediated killing by neutrophils [19, 20]. A similar report indicates that SPE B degrades C3 and contributes to escape of GAS from innate immunity at the site of infection using *in vivo* infection model [21]. Honda-Ogawa *et al*. reported that SPE B degrades the assembled membrane attack complex C5b-C9 and complement regulator C1-esterase inhibitor, which contributes to increasing GAS survival in human serum [22]. These results indicate that SPE B helped bacteria to resist the complement defense system and allowed the GAS to multiply.

In the present study, we further indicated that SPE B binds to human serum C3 through its C-terminal domain, and the major C3-binding motif is located between residues 376 and 398 in SPE B. Furthermore, immunization with a 23mer synthetic peptide that consisted of residues 376–398 of SPE B protected mice from a lethal GAS invasive infection.

## Materials and Methods

### Bacteria

The M49 GAS strain NZ131 was a gift from Dr. D. R. Martin, New Zealand Communicable Disease Center, Porirua. Two clinical M1 GAS strains A1 and A20, isolated from culture of blood from patients with necrotizing fasciitis in National Cheng Kung University Hospital, were kindly provided by Dr. J. J. Wu (Department of Medical Laboratory Science and Biotechnology, NCKU). Cultivation and quantification of bacteria were carried out as previously described [23].

### Cloning, expression and purification of recombinant truncated SPE B mutants

The genomic DNA of M1 GAS strain A20 was extracted and the *spe*B gene was amplified by PCR to produce the inactive SPE B point mutant C192S as described previously [24]. DNA sequences encoding several SPE B truncated mutants were amplified by PCR using the pET-21a plasmid, which contained the C192S mutant as template. Five pairs of specific primers with *Bam*H1 and *Xho*1 restriction sites for making the SPE B truncated mutants are listed in Table 1. The PCR products were purified and then cloned into the *Bam*H1 and *Xho*1 restriction sites of the pET-42a vector. The recombinant plasmids were transformed into *Escherichia coli* BL21(DE3) pLyS strains, and the recombinant His-tagged, glutathione S-transferase (GST)-conjugated SPE B mutant proteins were produced by growing the transformants in LB broth containing kanamycin (50 μg/ml) at 37°C. The expression of recombinant proteins was induced by adding of 0.5 mM isopropyl-β-D-thiogalactopyranoside and cells were grown at 37°C for a further 4 h. The cells were harvested by centrifugation and lysed by sonication, the lysate was centrifuged at 12,000 × g for 15 min, and the supernatant was filtered through a 0.45-μm filter. SPE B truncated mutants were purified using Ni^2+^-chelating column (GE Healthcare), and the purified recombinant proteins were further examined using 12% sodium dodecyl sulfate-polyacrylamide gel electrophoresis (SDS-PAGE) and confirmed by Western blotting using rabbit anti-SPE B serum as previously described [25]. The C-terminally truncated SPE B mutant SPE B_146–268_ and N-terminally truncated SPE B mutant SPE B_269–398_ were kindly provided by Dr. W. Huang (Department of Medical Laboratory Science and Biotechnology, NCKU).

**Table 1:** Primers and peptides used in this study.

Primer Name	Sequence
*spe*B_146–319_-F	5′-CGCGGATCCCAACCAGTTGTTAAATCT-3′
*spe*B_146–319_-R	5′-CCGCTCGAGAATTTGTGCTTCCCAATC-3′
*spe*B_146–358_-F	5′-CGCGGATCCCAACCAGTTGTTAAATCT-3′
*spe*B_146–358_-R	5′-CCGCTCGAGACCCCAGTTAACATGGTA-3′
*spe*B_146–398_-F	5′-CGCGGATCCCAACCAGTTGTTAAATCT-3′
*spe*B_146–398_-R	5′-CCGCTCGAGAGGTTTGATGCCTACAAC-3′
*spe*B_281–358_-F	5′-GCGGATCCGGTTCTGCAGGTAGCTCTC-3′
*spe*B_281–358_-R	5′-CCGCTCGAGACCCCAGTTAACATGGTAGA-3′
*spe*B_345–398_-F	5′-GCGGATCCGATGGTGCTGACGGACG-3′
*spe*B_345–398_-R	5′-CCGCTCGAGAGGTTTGATGCCTACAACA-3′

Peptide Name	Sequence
PP1	SVHQINRGDFSKQDW
PP2	EAQIDKELSQNQPVY
PP3	YQGVGKVGGHAFVID
PP4	GADGRNFYHVNWGWG
PP5	NWGWGGVSDGFFRLDALNPS
PP6	ALGTGGGAGGFNGYQSAVVGIKP
SLS_PP_	FSIATGSGNSQGGSGSYTPGK
control peptide	GSGNLPGPPHGPRGQ

### Peptide synthesis

The synthetic peptides PP1 to PP6, which contain the C-terminal region from residues 301 to 398 of SPE B, and SLSpp, which contains amino acid residues 33 to 53 of streptolysin S, were synthesized by Genemed Biotechnologies, Inc. All synthesized peptides were purified using high performance liquid chromatography to a purity greater than 95%. The sequences of the synthetic peptides used for this study are shown in Table 1. Additionally, to enhance the immunogenicity of the PP4, PP6, SLSpp, and control peptides, they were synthesized with an additional cysteine residue at the N terminus for conjugation to keyhole limpet hemocyanin.

### Enzyme-linked immunosorbent assay (ELISA)

The wells of microtiter plate (NUNC) were coated with 50 μl of equal moles of the different SPE B recombinant proteins, including the point mutant C192S and the SPE B truncated mutants at 37°C for 1 h. The plates were washed and then blocked with 200 μl of 1% bovine serum albumin (BSA)/PBS at 4°C overnight. After washing, fifty microliters of PBS-diluted human sera (1:1000) were added to the plates and incubated at 4°C overnight. Unbound serum C3 was washed out by 0.1% Tween-20 in PBS (PBS-T). Anti-human C3 polyclonal antibody (Calbiochem) (1:5000) was added and incubated for 1 h at 37°C. After washing, peroxidase-conjugated rabbit anti-goat IgG antibody (Calbiochem) (1:10000) was added to the wells for 1h at 37°C. Next, the 3, 3′, 5, 5′-tetramethylbenzidine (TMB) substrate (Vector Laboratories) was added, and absorbance values were read at 650 nm. In each assay, the maximal absorbance value of the C192S mutant bound to serum C3 was set as 100%. The relative binding activity of each recombinant protein to serum C3 was calculated as follows: binding activity = 100% × (*A*_*650*_ (recombinant SPE B protein)) / (*A*_*650*_ (C192S protein) [25]. In the C3-peptide binding assay, the ELISA plates were coated with 0.5 μg of synthetic peptides instead of the SPE B recombinant proteins for the C3-binding motif assay.

### Competitive binding inhibition assay

Microtiter plates were coated with 1 μg of the SPE B point mutant C192S in 50 μl of coating buffer at 37°C for 1 h. The plates were washed and then blocked with 1% BSA in PBS. The PBS-diluted human sera (1:1000) were incubated with 1 or 10 μM of the control peptides, synthetic peptides PP4, PP5, PP6 or recombinant mutant SPE B_345–398_ at 37°C for 30 min and these mixtures were then added to the C192S-coated plates and incubated at 4°C overnight. After several washes with 0.1% PBS-T, the anti-human C3 polyclonal antibody (1:5000) and peroxidase-conjugated rabbit anti-goat IgG antibodies (1:10000) were added sequentially, and then developed with TMB substrate at 650 nm as described above. In each assay, the maximal absorbance value of the C192S mutant bound to serum C3 was set as 100%. The percent inhibition of synthetic peptides or SPE B_345–398_ was calculated as follows: 100% × [1- (*A*_*650*_ (serum incubated with synthetic peptides or SPE B_345–398_))/ (*A*_*650*_ (serum without incubation with synthetic peptides or SPE B_345–398_)).

### GST pull-down assay

The *in vitro* interaction between the GST-tagged recombinant SPE B mutants and human C3 were assessed using GST pull-down assays. Equal moles of SPE B recombinant proteins, including SPE B_146–319,_ SPE B_146–358_ and SPE B_146–398_, were gently mixed with 1:20-diluted human serum in binding buffer (20 mM HEPES pH 7.4, 150 mM NaCl, 0.5 mM EDTA, 1 mM DTT, 10% Glycerol, 0.1% Triton X-100) at 4°C for 0.5 h. Then, fifty microliters of glutathione-sepharose beads that were pre-equilibrated in TEE buffer (50 mM Tris, pH 7.4, 1 mM EDTA, 1 mM EGTA) were added into the serum-SPE B mixtures and gently mixed at 4°C for 2 h. The beads were collected and washed 5 times with TEE buffer containing 1% Triton X-100 and then suspended in 25 μl of 2X SDS loading buffer and boiled for 5 min. The bound complexes were separated by 12% SDS-PAGE and analyzed using Western blotting with goat anti-human C3 antibody.

### Mice and immunizations

Seven- to 8-week old male BALB/cByJNarl mice were used in all experiments, which were purchased from the National Laboratory Animal Center in Taiwan. The mice were maintained on standard laboratory chow and water ad libitum in the animal center at I-Shou University. The synthetic peptides (1 mg/ml) were mixed with an equal volume of Freund’s complete adjuvant (Sigma-Aldrich), and 0.1 ml of the emulsion was inoculated intraperitoneally into the mice under anaesthesia with 5% isoflurane inhalation. The mice then received 3 booster immunizations of the synthetic peptides (25 μg of each was emulsified with Freund’s incomplete adjuvant) at two-week intervals. The sera of the mice were collected under isoflurane anaesthesia, and the titer of the anti-SPE B antibody of each mouse was determined using ELISA, as previously mentioned [25]. After immunization, groups of 5–10 BALB/c mice were infected through the air-pouch route with 2~3 × 10^8^ colony forming units (CFU) of M49 strain *S. pyogenes* NZ131, M1 clinical strain A1 or M1 clinical strain A20. The infected mice were monitored twice a day, and mice survival rates as well as the tissue lesions were examined for a total of 14 days. During the observation time, the degrees of skin lesion were photographed and measured using the ImageJ software (National Institutes of Health, Maryland, USA) as previously described [25]. The skin tissues surrounding the lesions were collected from the immunized mice and control mice at 48 h after GAS infection for histopathological analysis after being sacrificed by carbon dioxide (CO_2_) inhalation. The air pouch exudates collected from the GAS-infected mice were analyzed using Western-blot analysis for the C3 levels with goat anti-human C3 antibody. During the experimental periods, the mice that manifested clinical signs, including dyspnea, cyanosis, complete anorexia, and 25% weight loss, were humanely sacrificed using CO_2_ euthanasia to avoid unnecessary suffering. At day 15 post-infection, all the mice were sacrificed by CO_2_ euthanasia and also received cervical dislocation to ensure death. All procedures, care and handling of the animals was reviewed and approved by the Institutional Animal Care and Use Committee at I-Shou University (IACUC-ISU-99026).

### Histopathology

Four days after the final booster injection, mouse serum was collected, and the levels of serum creatinine (CRTN) and blood urea nitrogen (BUN) were measured using the Beckman Image System (Beckman Coulter, Brea, CA, USA). The results of CRTN and BUN are presented as mg/dl. Skins, kidneys, and hearts from the control and immunized mice were collected for histopathologic analysis. The tissue samples were fixed in 10% neutral buffered formalin solution, embedded in paraffin, sliced into 4-μm-thick sections, and stained with hematoxylin-eosin (H&E).

### Statistics

The survival curves of mice were compared for significance using the log-rank test. Statistical analysis was performed using ANOVA. Differences were considered significant at *P* < 0.05.

## Results

### The C-terminal domain of SPE B bound human serum C3

**Figure 1:**
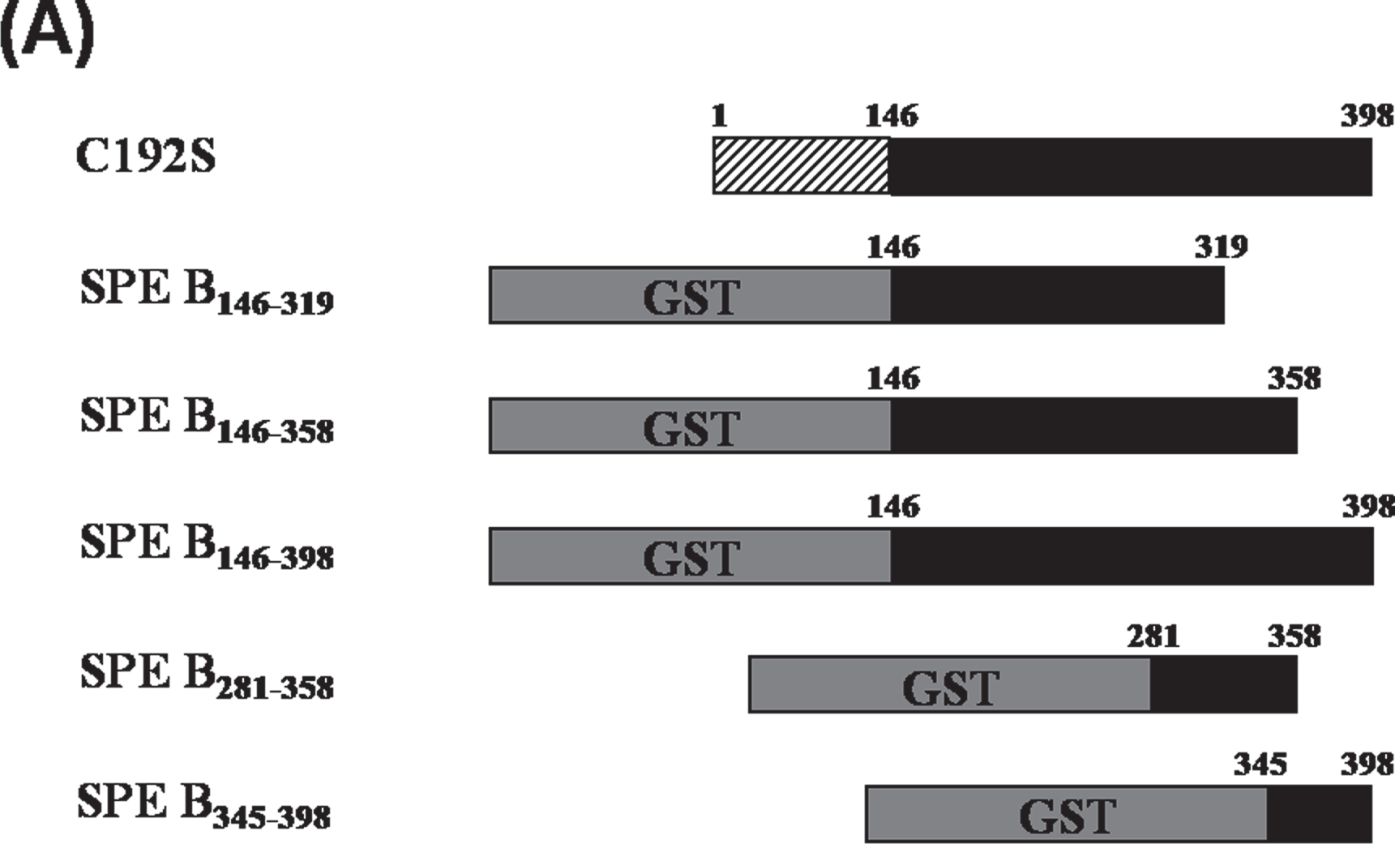
Truncated recombinant SPE B mutants and synthetic peptides. Schematic diagrams of different recombinant SPE B fragments, including SPE B_146–319_, SPE B_146–358_, SPE B_146–398_, SPE B_281–358_, SPE B_345–398_, and C192S. The cloning and expression of the different *speB* gene segments were described in the Materials and Methods. C192S, which contained a mutation at the active site Cys-192 of SPE B, represents a complete loss of protease activity mutant.

**Figure 2:**
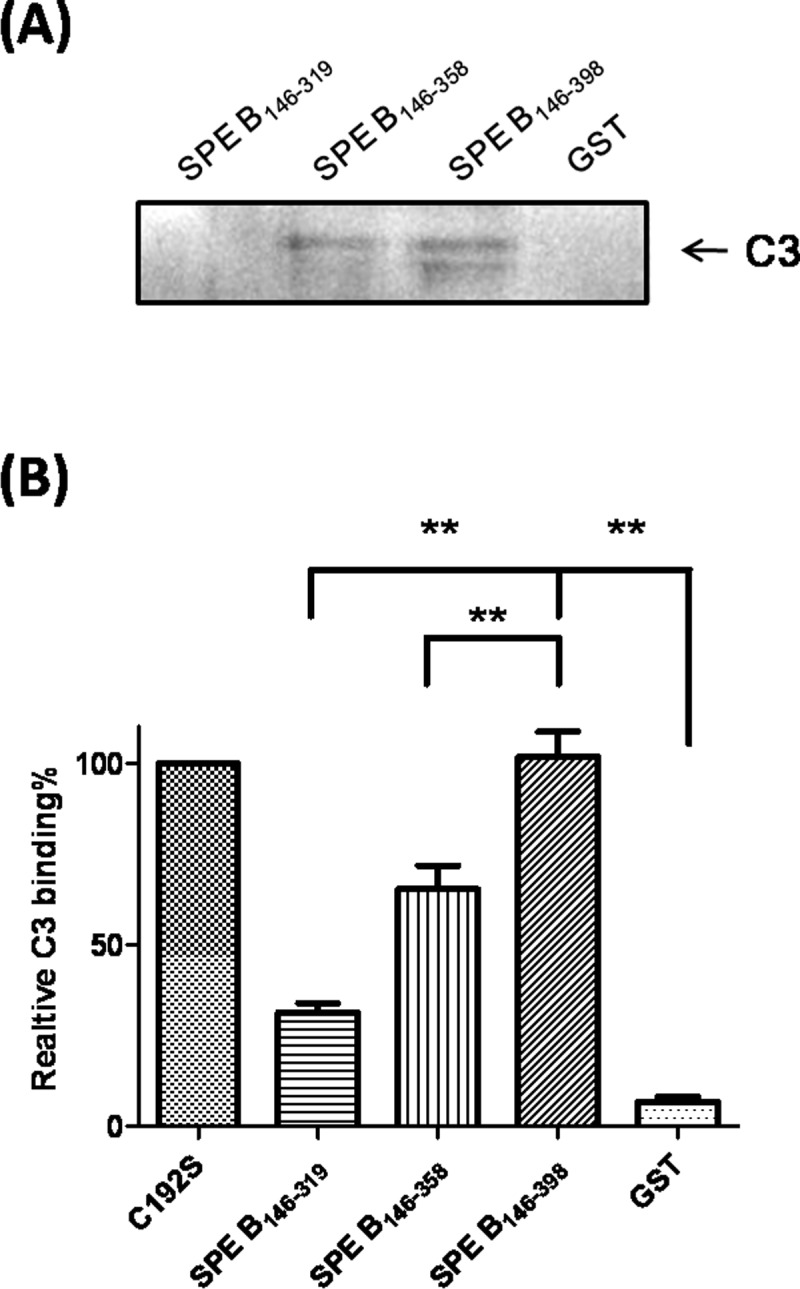
Binding of the SPE B fragments to human serum C3. (A) Immunoblot for complement C3 using eluents from GST-sepharose beads or different SPE B fragments conjugated the GST-sepharose beads and incubated with human serum. The reaction eluents were separated using 12% SDS-PAGE and blotted with goat anti-human C3 polyclonal antibody, as described in the Materials and Methods. (B) ELISA for binding of serum C3 to coated C192S, GST or SPE B fragments. The binding efficiencies were expressed as the ratios of C3-binding to the SPE B fragments to the binding of C3 to C192S, as described in the Materials and Methods. **P< 0.01, compared with values determined for SPE B_146–398._

To examine the human C3 binding domain of SPE B, a series of terminal truncated mutants of SPE B were constructed where the cysteine192 residue was replaced by serine to completely block the protease activity. The truncated mutant SPE B_146–398_ represented a mature form of SPE B (mSPE B) that lacks protease activity; SPE B_146–358_ and SPE B_146–319_ lack the C-terminal domain of mSPE B; and SPE B_281–358_ lacks the C-terminal and N-terminal domains of mSPE B (Fig. 1). The C3-binding activity of the C-terminal truncated SPE B mutants were determined using GST pull-down assays and ELISA. When comparing the C3-binding efficiencies of the C-terminally truncated SPE B mutants by GST-pull down assays, we found that the human serum C3 presented in the SPE B_146–398_ and SPE B_146–358_ pull-down fractions, and no C3 was present in the SPE B_146–319_ or GST-bound fractions (Fig. 2A). The C3-binding motif of SPE B was further determined using the recombinant SPE B fragments and ELISAs. The results indicated that SPE B_146–398_ had the strongest binding activity toward C3 than the other SPE B fragments, and its C3-binding efficiency was similar to that of C192S (Fig. 2B). However, the recombinant mutant with a deletion of 40 residues of the C-terminal portion of SPE B, SPE B_146–358_, showed decreased binding affinity to C3. The SPE B_146–319_, which contains the N-terminal domain of the mature form of SPE B, had poor binding capacity to human serum C3 (Fig. 2B). The results of the ELISAs and pull-down assays both indicated that the recombinant SPE B_146–319_ mutant had lost its C3 binding activity. Moreover, the binding activity of the C-terminally truncated SPE B mutant, SPE B_146–268_, or the N-terminally truncated mutant, SPE B_269–398_, to C3 was also determined using ELISA. The results indicated that the mutant protein SPE B_269–398_, but not the C-terminally truncated mutant SPE B_146–268_, bound to human serum C3 as efficiently as the full-length point mutant C192S (S1 Fig.). These results suggest that the C-terminal domain located between residues 320 and 398 of SPE B contains the binding motif of human C3.

### The major C3-binding motif is located between amino-acid residues 376 and 398 of SPE B

**Figure 3:**
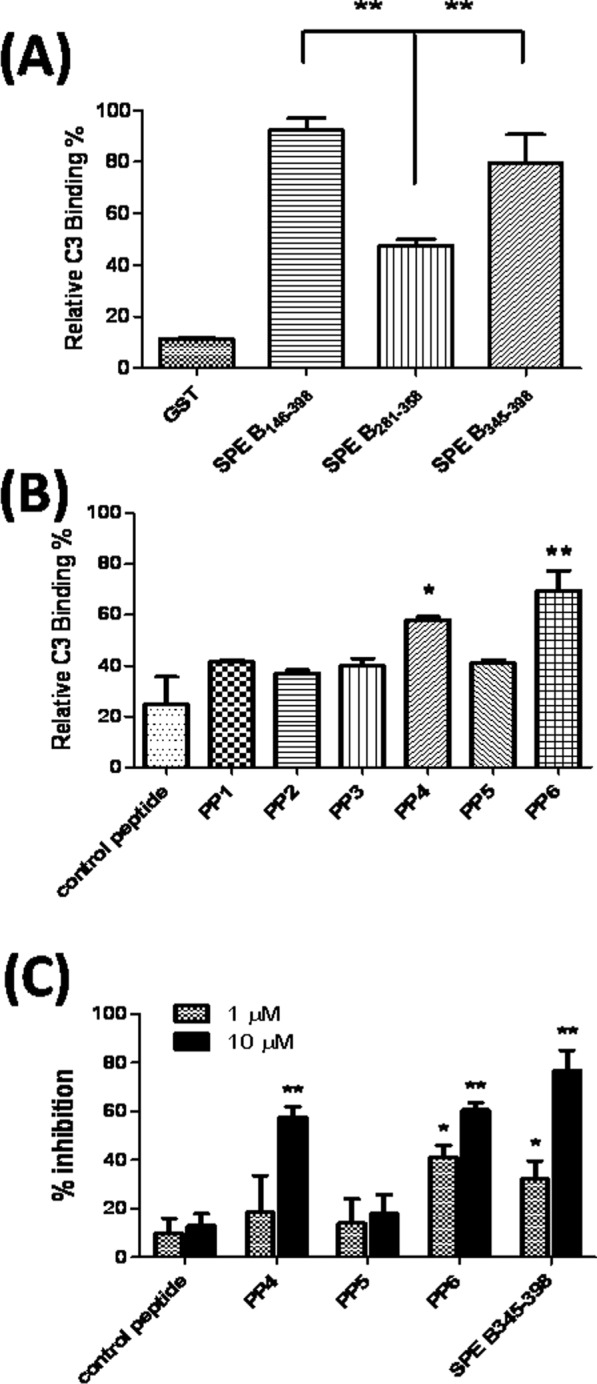
Serum C3 bound the C-terminal domain of SPE B. (A) Either equal moles of the purified recombinant SPE B mutant proteins, control GST or (B) the synthetic peptides PP1-PP6, containing the C-terminal region from residues 301 to 398, was used to coat a 96-well ELISA plate. The binding efficiency of the SPE B truncated mutants or synthetic peptides to serum C3 was detected by ELISA. (C) Different concentrations of synthetic peptides or SPE B_345–398_ were incubated with human serum and then added to the C192C-coated plates. The inhibiting ability of the synthetic peptides or SPE B_345–398_ was expressed as the ratio of C3-binding to the C192S with pretreatment of synthetic peptides or SPE B_345–398_ to the binding of C3 to C192S without pretreatment of synthetic peptides or SPE B_345–398_, as described in the Materials and Methods. The synthetic peptide PP4 and PP6 showed > 50% inhibitory activity on C3 and SPE B interaction. **P< 0.01, compared with values determined for the SPE B_281–358_ (A), *P< 0.05, **P< 0.01, compared with values determined for the control peptide (B, C).

We further constructed two recombinant SPE B_281–358_ and SPE B_345–398_ mutants to determine the C3 binding motif in the C-terminal domain of SPE B. The ELISA results indicated that the C3-binding efficiency of SPE B_281–358_ decreased; however, the binding affinity of SPE B_345–398_ to C3 was similar to that of SPE B_146–398_ (Fig. 3A). These results indicated that amino-acid residues 345 to 398 of SPE B are important for C3 binding. For precise mapping of the C3-binding sequence, six synthetic peptides (PP1 to PP6) that contained the C-terminal region from residues 301 to 398 of SPE B were examined using C3 binding assays (Table 1). The ELISA results indicated that the synthetic PP6 peptide, whose sequence region contains amino-acid residues 376–398 of SPE B, was the major C3-binding motif. Moreover, the synthetic PP4 peptide, whose sequence region contains amino acid 346 and 360, exhibited less C3-binding activity than PP6, but its C3-binding efficiency was higher than the others (Fig. 3B). For estimating the ability of C3-binding motifs of SPE B to interfere the interaction between C3 and SPE B, a competitive binding inhibition assay was performed. The competitive ELISA results shown that the levels of C3 binding to C192S was inhibited by synthetic peptides PP6 as well as the recombinant mutant SPE B_345–398_ at 1 and 10 μM concentrations. Furthermore, a 58% reduction in the C3 binding was observed after10 μM of synthetic peptides PP4–treatment serum, but it was only 14–18% reduction in the PP5 peptide-treatment serum (Fig. 3C). These results suggested two C3-binding motifs in the C-terminal domain of SPE B might exist.

### Immunization with the C3-binding motifs of SPE B protects mice from GAS infection

**Figure 4:**
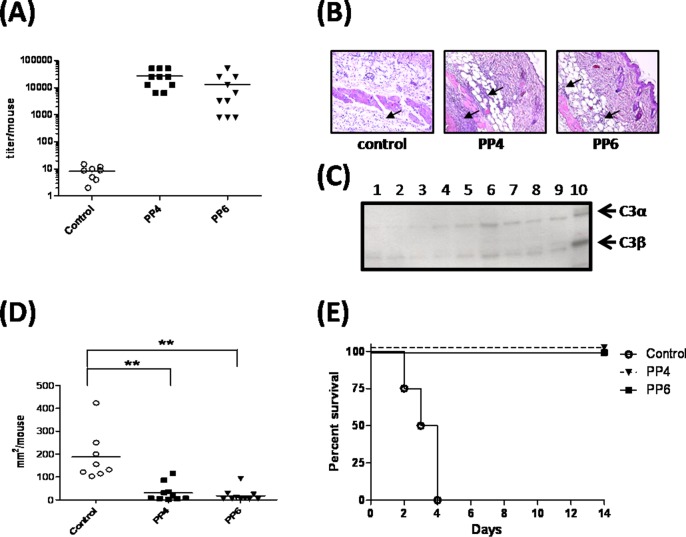
Immunization of synthetic peptides protects mice from M49 GAS strain-induced death. (A) Sera were collected from synthetic peptide PP4- or PP6-immunized mice, and the titers of anti-SPE B antibody were detected by ELISA. (B) Histopathological difference in control mice and peptide-immunized mice after infection with GAS. Groups of 8 to 10 BALB/c mice were immunized with synthetic peptides and inoculated via the air pouch route with 3 × 10^8^ CFU of the M49 GAS strain. Skins around the GAS-infected air pouch were excised, fixed, and stained with H&E reagent at 48 h post-infection. A large number of infiltrated cells were distributed around the connective tissues from the PP4- or PP6-immunized mice but not in tissues from GAS-infected control mice (arrows). (C) Levels of C3 in the air pouch exudates collected from control group (lane 1–3), PP4-immunized mice (lane 4–6) and PP6-immunized mice (lane 7–9) on day 2 after GAS infection. The degradation of C3 by GAS infection was analyzed by Western blot with anti-C3 antibody. Human serum dilute with PBS (1:100) was a positive control for C3 (lane 10). (D) The area of the skin lesions around the air pouch of the GAS-infected mice was estimated at 48 h post-infection. **P< 0.01, compared with the values determined for control group. (E) The survival rates in control or synthetic peptide-immunized mice after infection with M49 GAS. The survival curves were compared for significance using the log-rank test for the immunized mice versus the control group (*P*< 0.01).

In a previous report, immunization of BALB/c mice with the N-terminally truncated recombinant mutant SPE B_345–398_ protected the mice from lethal infection by M49 GAS strain NZ131 [25]. To examine whether the synthetic peptides corresponding to the C3-binding motif of SPE B could induce the production of antibodies and protect against lethal GAS infection, we immunized BALB/c mice with the PP4 or PP6 synthetic peptides, and the levels of anti-SPE B were determined using ELISA after the last boost. The synthetic peptide-immunized mice developed antibodies that reacted with C192S (Fig. 4A). A lethal dose of the M49 GAS (3 × 10^8^ CFU/mouse) was inoculated into the air-pouches of synthetic peptide-immunized mice or control mice. The tissues surrounding the air pouch were collected, formalin-fixed, paraffin-embedded at 48 h after GAS infection, and then examined by H&E staining. The H&E stained skin tissues from GAS-infected control mice showed sever necrosis of epidermis, dermis and subcutaneous fat, which did not present in the tissues from the GAS infected synthetic peptide-immunized mice. The numbers of infiltrating cells in inflamed skin tissues of the PP4- or PP6-immunized mice were much higher than tissues from control mice (Fig. 4B). The degradation of C3 was observed in the air pouch exudates of control mice at 48 h after GAS infection, but the degradation of C3 was less apparent in exudates from the PP4- or PP6-immunized mice (Fig. 4C). These results indicated the antibodies induced by synthetic peptides PP4 and PP6 could prevent the C3 cleavage by SPE B after GAS infection. Furthermore, the skin lesions of the synthetic peptides-immunized groups were significantly smaller than the control mice (Fig. 4D). All control mice died within 4 days, and all of the immunized mice survived 14 days after infection with the M49 GAS strain (Fig. 4E). These results indicated that immunization with the PP4 or PP6 synthetic peptides effectively protected a lethal dose of M49 GAS infection.

Previous reports suggest that production of SPE B and elevation of anti-SPE B antibody are both involved in the pathogenesis of post-streptococcal glomerulonephritis [26, 27, 28, 29]. In this study, we did not find characteristics of glomerulonephritis or myocarditis in the PP4- or PP6-immunized mice (S2 Fig.). The CRTN and BUN levels of the PP4-immunized group (CRTN: 0.43 ± 0.05 mg/dl; BUN: 20.2 ± 2.91 mg/dl) or PP6-immunized group (CRTN: 0.45 ± 0.06 mg/dl; BUN: 21.9 ± 2.84 mg/dl) were similar to those of the control group (CRTN: 0.41 ± 0.01 mg/dl; BUN: 19.5 ± 1.72 mg/dl). There were no statistical difference between the immunized groups and the control groups (*P* > 0.05). These results indicate that immunization with PP4 or PP6 was able to protect mice from GAS infection, but could not induce autoimmune-like symptoms.

**Figure 5:**
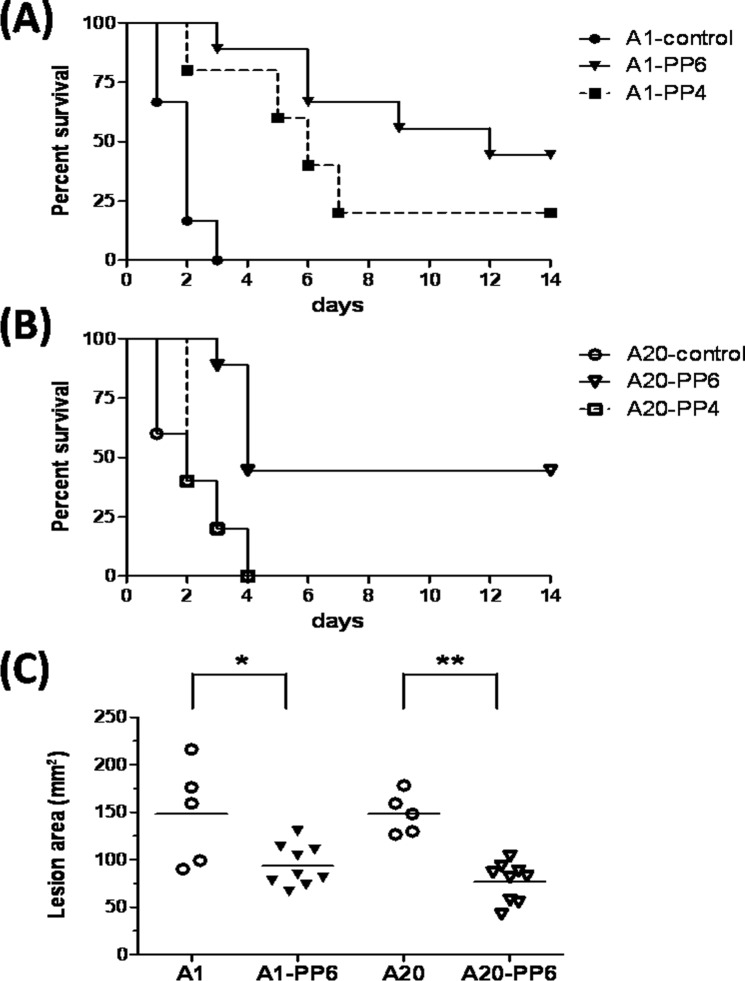
Immunization with the PP6 synthetic peptide partially protects mice from invasive GAS strains infection. The survival rates of the PP4 or PP6 synthetic peptide-immunized mice after infection with the clinically invasive A1 (A) or A20 GAS strains (B). Groups of 5 to 9 BALB/c mice were immunized with the control peptide (n = 5), synthetic peptide PP4 (n = 5) or synthetic peptide PP6 (n = 9) and inoculated via the air pouch route with 2× 10^8^ CFU of the A1 or A20 GAS strains. The survival curves were compared for significance using the log-rank test for the PP6-immunized mice versus the control group (*P*< 0.01). (C) The area of the skin lesions around the air pouch of the control (n = 5) or PP6-immunized mice (n = 9) at 48 h after A1 or A20 GAS strain infection. *P< 0.05, **P< 0.01, compared with the values determined for PP6-immunized groups.

The M49 GAS strain NZ131 was originally isolated from a clinical case of acute post-streptococcal glomerulonephritis, but not from an invasive GAS infection case. Therefore we chose two other M1 GAS (strain A1 and A20) isolates from patients with necrotizing fasciitis to evaluate the protective efficiency of immunization with the synthetic peptides. The control mice all died within 4 days after being inoculated with 2 × 10^8^ CFU of A1 or A20, and the survival rates of the PP6-immunized mice elevated to 44% in the A1- and A20-infected groups (Fig. 5A and 5B). In contrast to PP6, the PP4-immunized mice provided partial protection in A1-infected mice (Fig. 5A) while provided no protection in A20-infected mice (Fig. 5B). The skin lesions of PP6-immunized mice significantly decreased compared with the control mice after A1 or A20 GAS infection (Fig. 5C). These results indicated that immunization with the synthetic PP6 peptide, which corresponds to the major C3-binding motif of SPE B, provided nearly 45% protection against clinically invasive M1 GAS infection.

### Immunization of the synthetic peptides of SPE B and streptolysin S effectively protect mice from invasive M1 GAS infection

**Figure 6:**
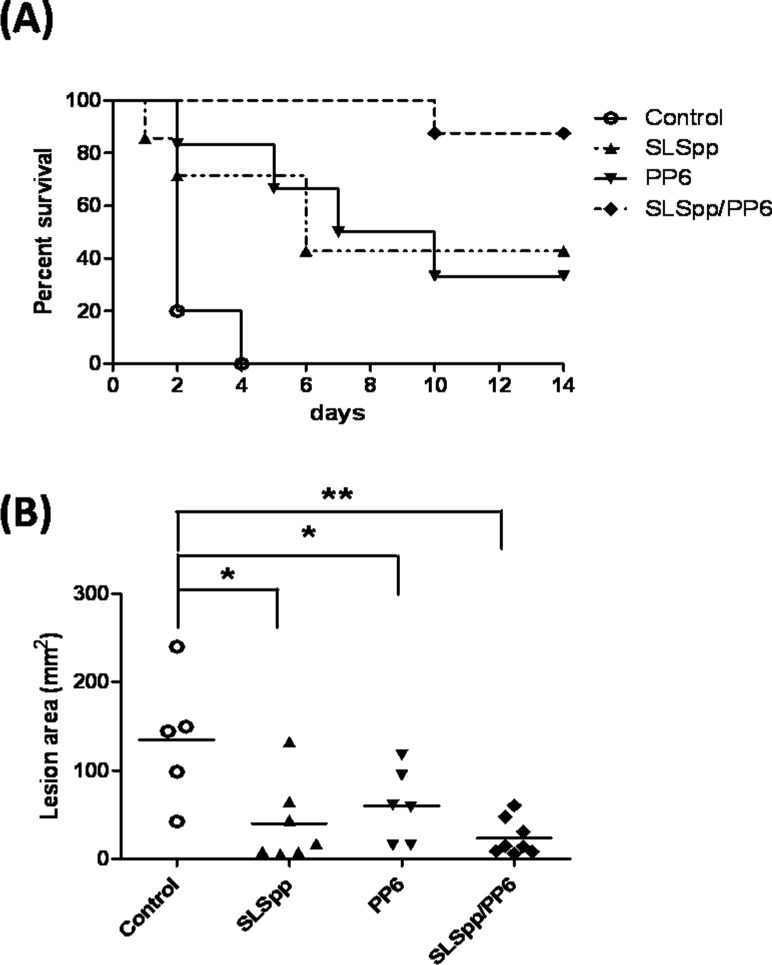
Immunization with the PP6 and SLSpp synthetic peptides protects mice from invasive M1 GAS infection. (A) The survival rates of synthetic peptide SLSpp, PP6, or a combination of two peptides (SLSpp/PP6)-immunized mice after infection with the clinically invasive GAS strain A1. Groups of 6 to 8 BALB/c mice were immunized with the SLSpp (n = 7), PP6 (n = 6) or SLSpp/PP6 synthetic peptides (n = 8) and subsequently inoculated via the air pouch route with 2× 10^8^ CFU of GAS strain A1. The survival curves were compared for significance using the log-rank test for the immunized mice versus the control group (*P*< 0.01). (B) The area of the skin lesions around the air pouch of the control or synthetic peptide-immunized groups 48 h after A1 GAS strain infection. *P< 0.05, **P< 0.01, compared with the values determined for the control mice.

Our previous study shows that SPE B and SLS have a synergistic effect on the pathogenesis of GAS invasive infection [30]. To achieve higher efficiency against invasive M1 strain GAS infection, a combination of PP6 with synthetic peptides derived from the C-terminal epitope of streptolysin S (SLSpp) was used to elicit a specific immune response toward streptococcal exotoxins SPE B and SLS. The death rates and the severity of skin lesions decreased significantly in SLSpp combined with PP6 peptide-immunized (SLSpp/PP6-immunized) mice compared with the control mice when challenged with a lethal dose of the M1 GAS strain (Fig. 6). The survival rates of the SLSpp/PP6-immunized mice were significantly improved to 88%, compared with 33% for the PP6-immunized group or 43% for the SLSpp-immunized group (Fig. 6A). Although the overall skin lesions were not significantly different between the synthetic peptide-immunized groups, a significant decrease in the SLSpp/PP6-immunized group was evident when compared with control mice (Fig. 6B). These results indicate that a combination of two synthetic peptides, whose sequences contain the C3-binding motif of SPE B and the C-terminal epitope of SLS, can be used to develop a subunit vaccine that protects against invasive M1 GAS infection.

## Discussion

SPE B, an important virulence factor of GAS, cleaves a number of host proteins that are important for the immune response against group A streptococcal infection [9]. Previous studies indicated that SPE B degrades several complement factors, including C1-esterase inhibitor, properdin, C3, C4, C6 and C9, to evade attack by the innate immune system [19, 20, 21, 22]. It also cleaves the Fc portion of serum IgG, which contributes to bacterial evasion from antibody-mediated opsonophagocytosis [31, 32]. These results indicate SPE B, with broad substrate-cleaving activity, can evade the host immune defense by interfering with the innate and adaptive immune systems.

Our previous study found the C-terminal domain amino-acid residues 345–398 of SPE B were the major binding site for human IgG [25]. In this study, we identified the C3-binding motif also located within C-terminal region of SPE B. Based on results of ELISA and GST-pull down assay, both showed that the truncated SPE B_146–358_ and SPE B_146–398_ proteins, but not the C-terminal truncated SPE B_146–319_ mutant, were able to bind with human serum C3 (Figs. 2 and 3A). For further characterizing the binding site of C3 on SPE B, six 15–23 residue-long peptide fragments of SPE B were synthesized and assessed for human serum C3-binding ability. The results of the ELISA indicated that two synthetic peptides whose sequences ranged from residue 346 to 360 and 376 to 398 displayed higher binding ability to serum C3 (Fig. 3B). These results indicated C-terminal amino acid residues 346 to 398 of SPE B were important for C3 binding, even though it is not the part of the catalytic domain of SPE B.

González-Páez and Wolan reported that the residues Ala^376^ and Gly^385^, which locate at the hinge position of C-terminal loop, control the loop movement to accommodate substrate binding, are important for controlling the accessibility of substrates to the catalytic site of SPE B [33]. In this study, we identified two C3-binding motifs both located within amino-acid residues 346–398 of SPE B. The synthetic peptide PP6 (Ala^376^-Pro^398^), contains amino acid residues of the glyicne-rich C-terminal loop of SPE B and effectively interferes with the interaction of C3 and SPE B at a concentration of 1μM. This major C3-binding motif (Ala^376^-Pro^398^) of SPE B contains these two key residues, Ala^376^ and Gly^385^, and play an important role to interfere with the complements activation (Fig. 3B). At a higher concentration (10 μM) of the synthetic peptide PP4 and PP6 both inhibited serum C3 binding to C192S up to 60% (Fig. 3C). Although the synthetic peptide PP4 (Gly^346^-Gly^360^) and PP5 (Asn^356^-Ser^375^) both contained residues Trp^357^ and Trp^359^ of SPE B, which previously reported for substrate binding [34, 35], but the C3-binding ability of PP4 was much higher than PP5 (Fig. 3B). These results indicated the other amino acids within residues 346 to 360 of SPE B were important for C3 and SPE B interaction. Many bacteria produce a number of proteins to interfere with complement system activation and evade the attacks of host innate immunity. Extracellular fibrinogen-binding protein (Efb), *Staphylococcus aureus* binder of immunoglobulin (Sbi) and Efb homologous protein (Ehp) are the C3-binding proteins produced from *S. aureus* [36, 37, 38, 39]. In spite of its low sequence identity, the synthetic peptide PP4 (Gly^346^-Gly^360^) contains Arg^350^, Val^355^, and Asn^356^, which are highly conserved in the C3-binding motifs (RxxxxVN) of Efb, Sbi and Ehp [39, 40]. Analysis of the C3-binding regions of those C3-binding proteins identified two conserved key residues, Arg^231^ and Asn^238^, that are important for the Sbi and C3 interaction [40]. However, the role of Arg^350^ and Asn^356^ of SPE B for C3-binding need further investigation.

In our previous study, we found that the recombinant SPE B_345–398_ protein could efficiently block cleavage of immunoglobulins by SPE B and interfere with SPE B-induced inhibition of complement activation. Immunization with SPE B_345–398_ protected mice from a lethal-dose infection of M49 GAS [25]. In the present study, immunization with the C3-binding motifs of SPE B efficiently protected mice against challenge with a lethal dose of M49 GAS strain and decreased the mortality from clinical invasive M1 strain GAS infection (Figs. 4 and 5). Patients infected with GAS may also develop immune-mediated post-infectious sequelae, such as rheumatic fever and post-streptococcal glomerulonephritis [3, 5]. Previous reports suggest that SPE B was involved in the pathogenesis of immune-mediated sequelae of GAS infection [26, 27, 28]. No significant histopathological changes were observed in the tissues of heart or kidney from the PP4- or the PP6-immunized mice (S2 Fig.). These results indicated that immunization with the synthetic peptides PP4 or PP6, which corresponded to the C3-binding motifs of SPE B Gly^346^-Gly^360^ or SPE B Ala^376^-Pro^398^, did not induce autoimmune-like symptoms.

The synthetic peptide PP6 that covers the last 23 C-terminal amino-acids of SPE B, which was distinct from previous identified autoepitope (amino-acid residues 296–310) of SPE B [41]. However, the protective efficiency of the PP6-immunination was not sufficient against invasive M1 strain GAS infection (Fig. 5). Our previous studies using an air pouch animal model shows that SPE B and SLS have a synergistic effect on the pathogenesis of GAS invasive infection [30]. Vaccination of C-terminal epitope of streptolysin S can inhibit SLS-mediated hemolysis and enhance M-protein antibody-mediated opsonophagocytosis [42].To achieve higher efficiency against invasive GAS infection, a combination of synthetic peptides derived from the C-terminal epitope of SLS (SLSpp) and peptides from the C3-binding motif of SPE B (PP6) was used to elicit a specific immune response against streptococcal exotoxins. The death rates and severity of skin lesions decreased significantly in PP6/SLSpp-immunized mice after infection with the invasive M1 GAS strain (Fig. 6). These results indicate the SPE B and SLS peptides could possibly be used to develop a combination subunit vaccine against invasive GAS lethal infection.

## Supporting Information

S1 Fig Deletion of the C-terminal domain of SPE B loss the binding activity to complement 3. (TIF)

S2 Fig Histological examination of hearts and kidneys from mice immunized with synthetic peptides. BALB/c mice were immunized with control peptide (A-B), synthetic peptide PP4 (C-D) or PP6 (E-F), and their hearts (A, C, E) and kidney (B, D, F) sections were stained with hematoxylin-eosin.
(TIF)

S1 ARRIVE Checklist. (PDF)
