# Supplementary figures and images for: Application of the C3-Binding Motif of Streptococcal Pyrogenic Exotoxin B to Protect Mice from Invasive Group A Streptococcal Infection
Source: PLoS One. 2015 Jan 28;10(1):e0117268. doi: 10.1371/journal.pone.0117268 (PMC4309557; doi:10.1371/journal.pone.0117268)

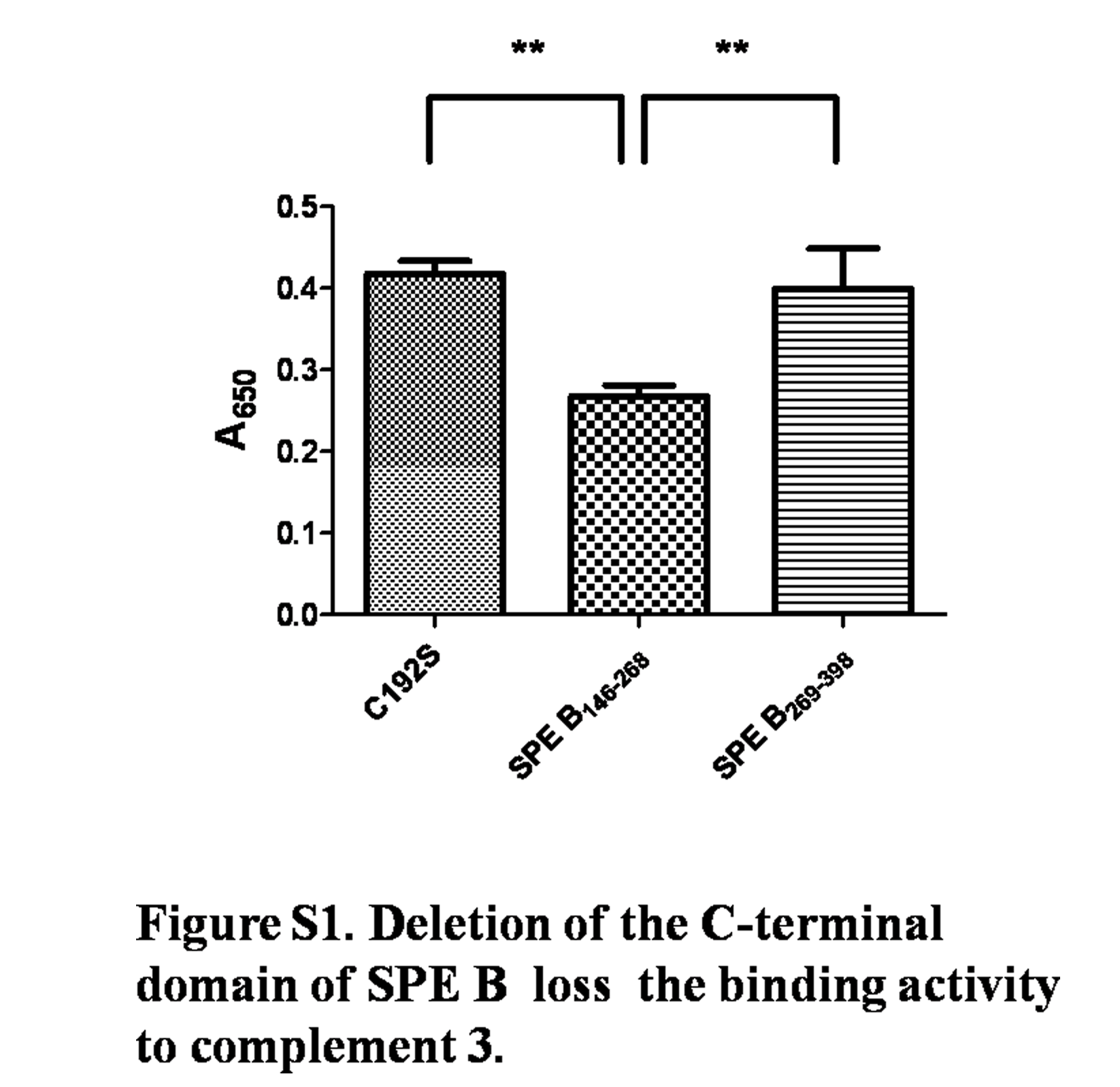

Supplement: S1 Fig — (TIF) [file pone.0117268.s001.tif]

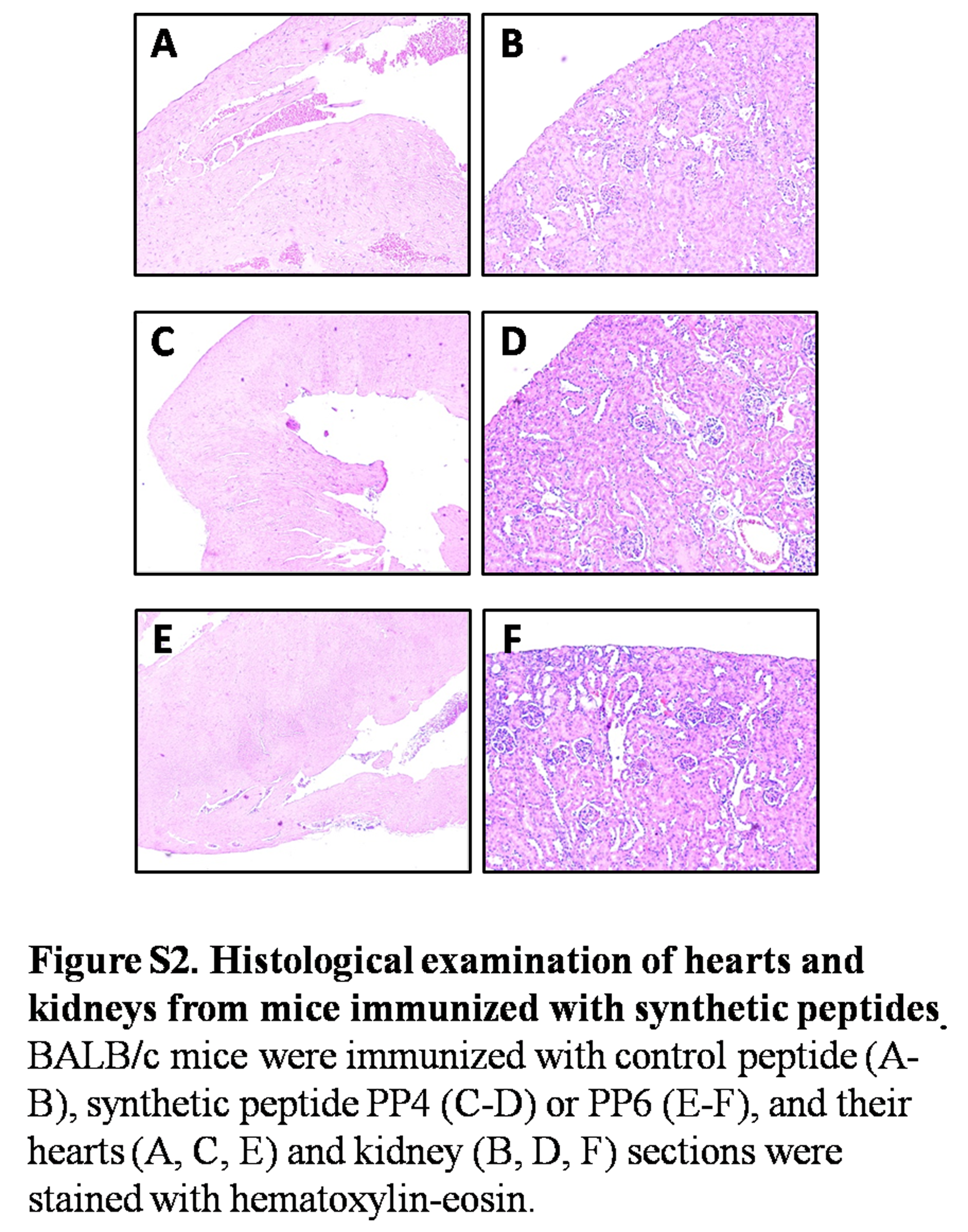

Supplement: S2 Fig — BALB/c mice were immunized with control peptide (A-B), synthetic peptide PP4 (C-D) or PP6 (E-F), and their hearts (A, C, E) and kidney (B, D, F) sections were stained with hematoxylin-eosin. (TIF) [file pone.0117268.s002.tif]
